# Application of Frequent Itemsets Mining to Analyze Patterns of One-Stop Visits in Taiwan

**DOI:** 10.1371/journal.pone.0014824

**Published:** 2011-07-01

**Authors:** Chun-Yi Tu, Tzeng-Ji Chen, Li-Fang Chou

**Affiliations:** 1 Institute of Hospital and Health Care Administration, School of Medicine, National Yang-Ming University, Taipei, Taiwan; 2 Department of Family Medicine, Taoyuan Veterans Hospital, Taoyuan, Taiwan; 3 Department of Family Medicine, Taipei Veterans General Hospital, Taipei, Taiwan; 4 Department of Public Finance, National Chengchi University, Taipei, Taiwan; University of Swansea, United Kingdom

## Abstract

**Background:**

The free choice of health care facilities without limitations on frequency of visits within the National Health Insurance in Taiwan gives rise to not only a high number of annual ambulatory visits per capita but also a unique “one-stop shopping”phenomenon, which refers to a patient' visits to several specialties of the same healthcare facility in one day. The visits to multiple physicians would increase the potential risk of polypharmacy. The aim of this study was to analyze the frequency and patterns of one-stop visits in Taiwan.

**Methodology/Principal Findings:**

The claims datasets of 1 million nationally representative people within Taiwan's National Health Insurance in 2005 were used to calculate the number of patients with one-stop visits. The frequent itemsets mining was applied to compute the combination patterns of specialties in the one-stop visits. Among the total 13,682,469 ambulatory care visits in 2005, one-stop visits occurred 144,132 times and involved 296,822 visits (2.2% of all visits) by 66,294 (6.6%) persons. People tended to have this behavior with age and the percentage reached 27.5% (5,662 in 20,579) in the age group ≥80 years. In general, women were more likely to have one-stop visits than men (7.2% vs. 6.0%). Internal medicine plus ophthalmology was the most frequent combination with a visited frequency of 3,552 times (2.5%), followed by cardiology plus neurology with 3,183 times (2.2%). The most frequent three-specialty combination, cardiology plus neurology and gastroenterology, occurred only 111 times.

**Conclusions/Significance:**

Without the novel computational technique, it would be hardly possible to analyze the extremely diverse combination patterns of specialties in one-stop visits. The results of the study could provide useful information either for the hospital manager to set up integrated services or for the policymaker to rebuild the health care system.

## Introduction

The people in Taiwan have free choice of healthcare providers and therapies within the universal National Health Insurance (NHI) program starting in 1995. The NHI provides comprehensive benefits in ambulatory care for western and Chinese medicines, dentistry, medications, laboratory examinations, surgeries, and inpatients services. The NHI and its contracted hospitals don't set a limit of physician visits within a day by a patient [1]. A patient can visit ambulatory clinics of any specialty in hospitals directly without formal referral and is only charged by a differential copayment [1], [2]. The great freedom in Taiwan's NHI caused not only a high number of annual visits per capita [1], but also a unique “one-stop shopping phenomenon” [2], which refers to visits of several specialties at the same facility in one day by a patient. A previous study estimated 7.6% of people in Taiwan had one-stop visits during a year [2].

Most literatures referring to one-stop shopping of health care focused on improving the service process through integrated settings. For example, the medical mall, developed in the USA in late 1980s, created a one-stop shop for ambulatory primary care, dental services, specialties for referral, pharmacy, physical therapy, government services, diagnostics, and other health care needs [3]. The integrated process for patients could save time intervals between their physician consultation, assessment, investigation and management [4]–[7]. Hospitals also provided additional services to their patients and visitors, with mall-like retail shops in or around their facilities, letting patients feel as at the mall [8].

The visits to multiple specialties, if not integrated, would increase not only the expense but also the potential risk of polypharmacy [9]. Although the percentage of persons with one-stop visits in Taiwan has been reported in a previous study [2], the patterns of one-stop visits needed to be further clarified. To our knowledge, there was no study on patterns of visits to multiple specialties in one day, especially for a large population.

In the current study, we analyzed a nationally representative claims dataset of 1 million people in Taiwan to calculate the frequency of one-stop visits. Because the one-stop shopping phenomenon is not rare in Taiwan and medical practice includes 24 specialties and 22 subspecialties, the number of combination patterns of specialties visited by patients would be hardly calculable with regular statistics or database programs. We thus adopted one of data mining techniques (the frequent itemsets mining) to compute the combination patterns of specialties in the one-stop visits. The analysis of the special case of one-stop visits in Taiwan will extend the application of data mining techniques to health services research.

## Methods

### Data sources

The single-payer NHI in Taiwan has covered almost all inhabitants (22,314,647 beneficiaries at the end of 2005, equaling 98.0% of all population). The NHI's electronic claims datasets, merged into a large computerized database as the National Health Insurance Research Database (NHIRD), have been released to the public for research purpose since 1999 (http://w3.nhri.org.tw/nhird//en/index.htm, accessed 2010 Feb 06). The database contains original claims for reimbursement plus registration files of beneficiaries and healthcare facilities. The identification numbers of persons and healthcare facilities in the datasets have been encrypted to protect privacy, but the encrypted identification numbers remain unique so that record-linking within datasets is feasible. All researchers who apply for use of the NHIRD are required to sign a written agreement declaring that they could not violate the privacy of patients or healthcare providers and should acknowledge the NHIRD on publication.

One special subset of NHIRD, the *Longitudinal Health Insurance Database for 2005* (LHID2005), contains all the registration and original claims data of 1,000,000 persons from 1996 to 2008. These 1,000,000 persons were randomly sampled from the 22,717,053 beneficiaries of the NHI program during the period from 1 January to 31 December 2005. According to NHIRD, there was no significant difference in the age, gender distribution and utilization between the people in the LHID2005 and those in the complete NHIRD datasets. Our analysis was limited to the year 2005.

### Study design

In 2005, the 1-million-person cohort had 15,037,163 ambulatory visit records. Only those with physician consultations of western medicine, dentistry, and traditional Chinese medicine were analyzed. We excluded the visits to emergency departments, prescription refills, home care by nurses, and preventive services without physician consultation.

One-stop visits in the current study were operationally defined as more than one visit for different specialties at the same healthcare facility on the same day by a patient. At first, the patients who had one-stop visits were extracted. The numbers and percentage of patients with one-stop visits in each age group and gender were calculated. We also compared them with the 1-million-person cohort.

The NHI in Taiwan divided medical specialties into 24 specialties and 22 subspecialties. The subspecialties, existing mostly at hospitals, were considered as different ones from their main specialties in our study. As to the combination patterns of specialties in one-stop visits, there would be theoretically 1,035 two-specialty combinations, 15,180 three-specialty combinations, 163,185 four-specialty combinations, and so on. If the number of visits in a day by a patient was not limited, the total kinds of possible specialty combinations would be about 7×10^13^. The ordinary statistical software programs don't provide a user-friendly and efficient solution without additional module or scripting. Besides, the statistical software is usually not suitable for processing a large amount of data. To overcome these difficulties, we adopted the frequent itemsets mining to tackle our research problem.

The frequent itemsets mining, a type of association rule mining, was developed in 1990s to analyze which groups of goods or sets of items were frequently purchased together. It has been used extensively in commercial marketing [10]–[12]. The most important concept of frequent itemsets mining is “inimal support” Among all items bought by a customer in one transaction there can be many subsets of items (itemsets), i.e. many possible combinations of individual items. If an itemset is repeatedly purchased with the frequency not less than the minimal support, then it is marked as a frequent itemset. The preset minimal support enables efficient computing of large-scale data. We have applied such a data mining technique to analyze the Taiwan's NHI claims databases in previous researches [13]–[15].

In the current study, the one-stop visits made by a patient to the same facility in one day correspond to items of one transaction in frequent itemsets mining. We set the minimal support for visit frequency at different levels iteratively, from 1000, 500, 100 and so on, to obtain frequent combinations of specialties (itemsets) in one-stop visits. The frequent combinations of specialties would be stratified by number of specialties (number of items in an itemset) in the final display.

### Data processing and statistical analysis

The open-source software Perl (version 5.10.0) (http://www.activestate.com/activeperl/, accessed 2010 March 3) was used for computing. The module of Data::Mining::AssociationRules version 0.1 by Dan Frankowski (http://search.cpan.org/dfrankow/Data-Mining-AssociationRules-0.10/, accessed 2010 March 3) was modified and integrated into the programming script. The regular statistics were displayed. In calculating the percentages of patients, the denominator was 1,000,000 according to the registry for beneficiaries in 2005.

## Results

There were 504,183 women and 495,817 men among the 1,000,000-person cohort in 2005. The ambulatory care visits with physician consultation amounted to 13,682,469 visits, and 79,371 (7.9%) persons did not have any visit during the year. The average number of visits per person was 13.7 (SD 14.6) in 2005. Nearly one fourth (n = 3,342,907) of visits were made to outpatient departments of hospitals.

One-stop visits occurred 144,132 times and involved 296,822 visits (2.2% of all visits) by 66,294 (6.6%) persons. The percentage of the persons with one-stop shopping behavior increased with age after age 10 and reached to the highest in the age group ≥80 years as 27.5% (5,662 of 20,579). In general, women were more likely to have this behavior than men (7.2% vs. 6.0%) (Table 1).

**Table 1:** Distribution of patients with one-stop visits^a^ in 2005, stratified by age and sex.

	All Persons in the 1,000,000-Person Cohort		Numbers of Patients With One-Stop Visits		
Age Group	Female	Male	Female (%)	Male (%)	All Sex (%)
0–9	54,683	59,992	1,207 (2.2)	1,673 (2.8)	2,880 (2.5)
10–19	66,756	71,573	1,083 (1.6)	1,205 (1.7)	2,288 (1.7)
20–29	91,275	77,842	2,896 (3.2)	1,740 (2.2)	4,636 (2.7)
30–39	84,568	81,441	3,775 (4.5)	2,230 (2.7)	6,005 (3.6)
40–49	80,722	81,209	5,738 (7.1)	3,729 (4.6)	9,467 (5.8)
50–59	58,388	57,176	7,321 (12.5)	4,650 (8.1)	11,971 (10.4)
60–69	34,279	31,454	6,472 (18.9)	4,563 (14.5)	11,035 (16.8)
70–79	23,252	24,811	5,643 (24.3)	6,707 (27.0)	12,350 (25.7)
≥80	8,899	9,368	2,390 (23.3)	3,272 (31.7)	5,662 (27.5)
All Age	504,183	495,817	36,525 (7.2)	29,769 (6.0)	66,294 (6.6)

a One-stop visits were defined as more than one ambulatory care visits for different specialties at the same healthcare facility on the same day by a patient.

When we set the minimal support for visit frequency at 1000, only 41 two-specialty itemsets were found in all visits. Until the minimal support set at 100, one three-specialty itemset began to appear. When the minimal support set at 10, the four-specialty itemsets could be obtained. The largest number of specialties visited at the same facility in one day by a patient was six. The overall number of different combinations was 4,709 (Table 2).

**Table 2:** Numbers of specialty combinations (itemsets)^a^ for one-stop visits^b^ at different minimal supports for visit frequency of an itemset, stratified by number of specialties in an itemset.

	No. of Specialty Combinations				
Minimal Support for Visit Frequency	2-Specialty Itemsets	3-Specialty Itemsets	4-Specialty Itemsets	5-Specialty Itemsets	6-Specialty Itemsets
1,000	41	0	0	0	0
500	106	0	0	0	0
100	255	1	0	0	0
50	341	19	0	0	0
10	776	285	9	0	0
1	2,070	1,876	655	98	10

a Subsets of specialties visited by a patient at the same health care facility on the same day.

b One-stop visits were defined as more than one ambulatory care visits for different specialties at the same healthcare facility on the same day by a patient.

Among one-stop visits, the most frequently visited specialties were ophthalmology, internal medicine, and cardiology. These three specialties had accounted for 72,607 (24.4%) visits in all one-stop visits (Table 3).

**Table 3:** Numbers of visits and patients in all one-stop visits^a^ in 2005, stratified by consulted specialty (selected).

Specialty	No. of Visits (%, n^b^ = 296,822)	No. of Patients (n = 66,294)
Ophthalmology	27,060 (9.1)	14,697
Cardiology	24,769 (8.3)	11,733
Internal medicine	20,778 (7.0)	11,989
Orthopedics	19,711 (6.6)	12,143
Gastroenterology	18,831 (6.3)	10,833
Neurology	17,801 (6.0)	8,947
Dermatology	15,522 (5.2)	9,500
Otorhinolaryngology	14,368 (4.8)	9,645
Urology	14,352 (4.8)	7,928
Endocrinology	12,735 (4.3)	5,895
Surgery	12,351 (4.2)	8,777
Family medicine	12,249 (4.1)	8,102
Gynecology	11,945 (4.0)	8,556
Rehabilitation	9,923 (3.3)	6,050
Pulmonology	9,367 (3.2)	5,510
Psychiatry	8,851 (3.0)	4,136
Others	46,209 (15.6)	29,020

a One-stop visits were defined as more than one ambulatory care visits for different specialties at the same healthcare facility on the same day by a patient.

b The total visit counts of all one-stop visits.

As to the combinations of specialties in one-stop visits, internal medicine plus ophthalmology was the most frequent combination with a visited frequency of 3,552 times (2.5% of 144,132 times of one-stop visits), followed by cardiology plus neurology with a visited frequency of 3,183 times (2.2%). The two-specialty combinations (itemsets) with a visited frequency > = 1000 were selectively displayed in Table 4. The most frequent three-specialty combination was cardiology plus neurology and gastroenterology, but only with a visited frequency of 111 times (0.08%) (Table 5).

**Table 4:** Patterns of two-specialty combinations in one-stop visits^a^ in 2005 (selected).

Specialty Combination	Visited Frequency (%, n^b^ = 144,132)	No. of Patients
Internal medicine + Ophthalmology	3,552 (2.5)	2,047
Cardiology + Neurology	3,183 (2.2)	1,575
Cardiology + Gastroenterology	3,085 (2.1)	1,786
Internal medicine + Orthopedics	2,758 (1.9)	1,870
Cardiology + Ophthalmology	2,745 (1.9)	1,512
Cardiology + Endocrinology	2,636 (1.8)	1,070
Internal medicine + Surgery	2,523 (1.8)	1,884
Ophthalmology + Dermatology	2,319 (1.6)	1,573
Family medicine + Ophthalmology	2,260 (1.6)	1,466
Otorhinolaryngology + Ophthalmology	2,238 (1.6)	1,681
Cardiology + Orthopedics	2,108 (1.5)	1,304
Endocrinology + Ophthalmology	1,968 (1.4)	1,166
Orthopedics + Ophthalmology	1,944 (1.3)	1,290
Cardiology + Urology	1,912 (1.3)	997
Neurology + Gastroenterology	1,809 (1.3)	1,068
Ophthalmology + Neurology	1,788 (1.2)	1,085
Cardiology + Chest medicine	1,703 (1.2)	1,055
Internal medicine + Rehabilitation	1,514 (1.2)	971
Others	120,050 (83.3)	84,919c

a One-stop visits were defined as more than one ambulatory care visits for different specialties at the same healthcare facility on the same day by a patient.

b Total times of one-stop visits.

c One patient might have more than one different combinations.

**Table 5:** Patterns of three-specialty combinations in one-stop visits^a^ in 2005 (selected).

Specialty Combination	Visited Frequency (%, n^b^ = 144,132)	No. of Patients
Cardiology + Neurology + Gastroenterology	111 (0.08)	48
Cardiology + Neurology + Endocrinology	93 (0.06)	41
Cardiology + Endocrinology + Ophthalmology	77 (0.05)	51
Cardiology + Gastroenterology + Orthopedics	73 (0.05)	39
Internal medicine + Ophthalmology + Dermatology	72 (0.05)	46
Cardiology + Neurology + Ophthalmology	72 (0.05)	37
Cardiology + Ophthalmology + Orthopedics	68 (0.05)	48
Cardiology + Endocrinology + Gastroenterology	65 (0.05)	37
Cardiology + Neurology + Urology	63 (0.04)	37
Cardiology + Ophthalmology + Dermatology	62 (0.04)	41
Cardiology + Gastroenterology + Ophthalmology	61 (0.04)	38
Cardiology + Chest medicine + Gastroenterology	61 (0.04)	32
Orthopedics + Ophthalmology + Dermatology	60 (0.04)	40
Cardiology + Gastroenterology + Urology	60 (0.04)	40
Ophthalmology + Dermatology + Neurology	56 (0.04)	32
Others	9,308 (6.46)	7,286^c^

a One-stop visits were defined as more than one ambulatory care visits for different specialties at the same healthcare facility on the same day by a patient.

b Total times of one-stop visits.

c One patient might have more than one different combinations.

## Discussion

The association rule mining, also known as market basket analysis, has been used to analyze the large database for the commercial marketing for more than one decade [11]. Our research team had successfully applied this data mining technique in medical researches, especially to analyze the co-prescriptions of drugs. We had identified the drugs prescribed in combination with antacids from the Taiwan NHI claims [13]. We had also analyzed the prescription patterns of Chinese herbal medicine for patients with allergic rhinitis [14], and chronic hepatitis [15] in Taiwan. In the current study, we further extended the frequent itemsets mining, a type of association rule mining, to the study of patient's behavior. Our approach offered another novel application of this data mining technique.

The possible reasons of the one-stop shopping phenomenon in Taiwan would be as follows: First, the NHI and its contracted hospitals don't set a limit of physician visits within a day by a patient. Direct visits to any specialty clinic in hospitals without formal referral are only charged by a differential copayment. Second, the hospitals are very easily accessible in this densely populated island country. They generally provide varieties of specialties in their outpatient departments and advanced medical equipments in ambulatory cares. It is very convenient for the patients to have different kinds of medical services at one site in a day. Third, the number of people with multimorbidity might increase in Taiwan's aging society. The current practice of medicine in Taiwan is fragmented into many subspecialties without integration and the multimorbid patients might not be treated efficiently.

Our study revealed that the elderly were more likely to have one-stop shopping behavior and the percentage was above 25% in those aged 70 and over. Multimorbidity in the elderly might be the major reason. Before age 70, the percentage of one-stop shopping among men was lower than that among women. Perhaps men tended to delay health help-seeking [16]. After age 70, the gender disparity reversed. Because of shorter life expectancy, men might have poorer health at the late stage of life.

If one patient could choose any number of 46 specialties of the same healthcare facility in a day, there would be theoretically a hundred trillion of combination patterns. In reality, the time constraint prevents a patient from visiting too many specialties in one day. In the current study, the greatest number of visited specialties in a one-stop shopping was only six, far fewer than the number of items held in a shopping basket of a supermarket. The number of combination patterns in our study was correspondingly fewer than that in commercial market basket analyses.

In the current study, ophthalmology was the most frequently visited specialty in one-stop visits, although it was only at the 7th place among all ambulatory visits in Taiwan [4]. A study of multimorbidity in Sweden also found that comorbidity occurred beyond chance and the most frequent pattern of co-existing diseases was visual impairment with heart disease [17]. The comorbidity of the visual problem with other diseases reflected the prevalence of diseases among specific patient groups at the demand side in Taiwan. On the other hand, the underlying reason might be at the supply side in that the ophthalmologist's techniques could not be easily substituted by other specialists and the hospitals offered more advanced equipments than practicing ophthalmologists.

As comorbidity usually increased with age and in patients with chronic conditions, the other top frequently visited specialties in one-stop visits for cardiology, internal medicine, orthopedics, gastroenterology, and neurology were compatible with the chronic conditions usually seen in the elderly [18], [19]. The otorhinolaryngology in the 7th place might just reflect the high service volume of this specialty in Taiwan's ambulatory care for diseases of the respiratory system [2].

As for the combination patterns, the top ones with minimal support of 1000 might reflect the frequent comorbid conditions in patients with one-stop visits. Many combinations, such as those between cardiology, neurology, gastroenterology and endocrinology, could be substituted by family medicine or internal medicine to decrease the fragmentation of care. On the other hand, some combinations might reflect the necessity of different specialties' management, such as internal medicine, cardiology, endocrinology, neurology, otorhinolaryngology or family medicine plus ophthalmology, orthopedics, surgery, urology or rehabilitation. These combinations could be considered into integrated care clinics if the healthcare facilities intend to improve the service process and patient satisfaction.

In the current study, the patterns of specialty combinations in one-stop visits were extremely diverse and the number of visits in each combination, even the most frequent one, was relatively low. The practical meaning is that it would be difficult for hospitals to offer customized, integrated, and efficient services under circumstances of low economies of scale. The one-stop visits are indeed convenient to patients who need various specialties to treat their problems. But the risk of induced demand and polypharmacy deserves further attention. The NHI in Taiwan has to establish a more regulated referral system with family physicians as health gatekeepers, especially for those with multiple chronic diseases.

## Limitations

Our study has some limitations. First, the income and residence of beneficiaries were not available in the NHIRD for privacy protection. The influence of socioeconomic factors on one-stop visits couldn't be explored. Second, the claims didn't contain data about the referral. We couldn't differentiate whether the one-stop visits were initiated by physicians' referral in the same healthcare facility or out of patients' free choice. Third, our study design might overlook the influence of disease types. In the NHI, only three diagnoses could be coded in one ambulatory visit. The coding was undertaken by physicians for administrative purpose and seldom verified. Fourth, our study was limited to specialties visited in one day. If a patient visited several specialties of the same healthcare facility in a short period of days, it might otherwise imply the inefficiency of managerial operation. Finally, because the phenomenon of one-stop visits is a special, if not unique, case of health care in one specific country (Taiwan), our data-mining approach can be applied to other countries only when a similar environment without strict regulation on patient access to health care exists and extensive datasets are openly available to analysis.

### Conclusions

With the data mining technique, we had successfully analyzed the diverse patterns of specialty combinations in one-stop visits from large-scale datasets, what was hardly feasible with an ordinary statistical method. Our approach demonstrated another novel application of frequent itemsets mining to the research of patients' behaviors. The results could provide useful information either for the hospital manager to set up integrated care of health service and for the policymaker to rebuild the health care system.
